# A comparative analysis of the outcome of malaria case surveillance strategies in Sri Lanka in the prevention of re‐establishment phase

**DOI:** 10.1186/s12936-021-03621-5

**Published:** 2021-02-09

**Authors:** W. M. Kumudunayana T. de A. W. Gunasekera, Risintha Premaratne, Deepika Fernando, Muzrif Munaz, M. G. Y. Piyasena, Devika Perera, Rajitha Wickremasinghe, K. D. N. Prasad Ranaweera, Kamini Mendis

**Affiliations:** 1 Anti Malaria Campaign, 555/5 Public Health Building, Narahenpita, Sri Lanka; 2 World Health Organization Regional Office for South-East Asia, New Delhi, India; 3 Department of Parasitology, Faculty of Medicine, University of Colombo, 25 Kynsey Road, Colombo, Sri Lanka; 4 Department of Public Health, Faculty of Medicine, University of Kelaniya, P.O. Box 6, Thalagolla Road, 11010 Ragama, Sri Lanka

**Keywords:** Malaria case surveillance, Prevention of re-establishment of malaria, Passive case detection, Active case detection, Reactive case detection, Proactive case detection, Yield, Malaria in Sri Lanka, Travel cohorts, Spatial cohorts

## Abstract

**Background:**

Sri Lanka sustained its malaria-free status by implementing, among other interventions, three core case detection strategies namely Passive Case Detection (PCD), Reactive Case Detection (RACD) and Proactive Case Detection (PACD). The outcomes of these strategies were analysed in terms of their effectiveness in detecting malaria infections for the period from 2017 to 2019.

**Methods:**

Comparisons were made between the surveillance methods and between years, based on data obtained from the national malaria database and individual case reports of malaria patients. The number of blood smears examined microscopically was used as the measure of the volume of tests conducted. The yield from each case detection method was calculated as the proportion of blood smears which were positive for malaria. Within RACD and PACD, the yield of sub categories of travel cohorts and spatial cohorts was ascertained for 2019.

**Results:**

A total of 158 malaria cases were reported in 2017–2019. During this period between 666,325 and 725,149 blood smears were examined annually. PCD detected 95.6 %, with a yield of 16.1 cases per 100,000 blood smears examined. RACD and PACD produced a yield of 11.2 and 0.3, respectively. The yield of screening the sub category of travel cohorts was very high for RACD and PACD being 806.5 and 44.9 malaria cases per 100,000 smears, respectively. Despite over half of the blood smears examined being obtained by screening spatial cohorts within RACD and PACD, the yield of both was zero over all three years.

**Conclusions:**

The PCD arm of case surveillance is the most effective and, therefore, has to continue and be further strengthened as the mainstay of malaria surveillance. Focus on travel cohorts within RACD and PACD should be even greater. Screening of spatial cohorts, on a routine basis and solely because people are resident in previously malarious areas, may be wasteful, except in situations where the risk of local transmission is very high, or is imminent. These findings may apply more broadly to most countries in the post-elimination phase.

## Background

Surveillance is a core intervention in malaria elimination programmes, and is all the more important after elimination in preventing the re-establishment of malaria, as stated in the World Health Organization (WHO) Global Technical Strategy for Malaria 2016–2030 [1]. In these situations, two case surveillance strategies are recommended by the WHO [2], Passive Case Detection (PCD), which is the detection of malaria cases among people who seek health care on their own usually for fever; and Active Case Detection (ACD), which, as the name implies involves searching actively for malaria infections, such as in people or populations at high risk but who may or may not be obviously ill [2]. Each of these case surveillance strategies requires a different component of the health system to operate, have different demands on the system and on human resources, and may produce very different yields and outcomes, depending on the circumstances.

Since eliminating malaria in 2012, the Anti Malaria Campaign (AMC), the national malaria programme within the Ministry of Health of Sri Lanka has sustained an effective ‘Prevention of Re-establishment” (POR) programme for malaria founded on a rigourous clinical, parasitological and entomological surveillance and response system, which operates throughout the country [3–5]. This was required on account of the high degree of receptivity in some parts of the country, which may, in fact, have increased by the recent introduction into the country of *Anopheles stephensi*, an efficient urban vector of malaria in other neighbouring countries [6]. The steady rate of importation of malaria infections into the country conferring a high degree of vulnerability adds to the risk of malaria re-establishment [3, 4]. Since eliminating malaria in 2012, all reported malaria cases in Sri Lanka have been imported cases except for a single case of Introduced malaria (a case contracted locally, with strong epidemiological evidence linking it directly to a known imported case, as defined by the WHO) in 2018 [4].

The case surveillance system in Sri Lanka post-elimination has been based on both passive and active case detection. Two types of active case detection are used, Reactive and Proactive Case Detection. Reactive Case Detection (RACD) is conducted in response to an index case of malaria in people who were exposed to the same risk as the index case or in people who might be at risk of contracting the disease from the index case. Proactive case detection (PACD) is screening high risk populations not prompted by a case of malaria (Table 1).

**Table 1:** Malaria case surveillance strategies deployed in Sri Lanka

Case Surveillance strategy		Definition	Examples of populations tested
1.	Passive Case Detection (PCD)	Detection of malaria cases among people who seek health care on their own usually for fever.	Patients seeking health care in the public and private health sectors e.g. General Practitioners, at tertiary care hospitals, clinics, and even laboratories.
2.	Reactive Case Detection (RACD)	Case surveillance conducted in response to an index case of malaria.	
	RACD travel-cohorts	Screening people who were exposed to the same risk of infection as the index case. i.e. travel contacts (identified through contact tracing)	Groups of pilgrims returning from India, the middle east; Gem traders, fisherman returning from Africa; Refugees from an endemic country.
	RACD spatial-cohorts (in neighbouring households*)	Screening people (resident non-travellers) who might have contracted the disease from the same source as the index cases (if it is an indigenous or introduced case) or be at risk of contracting the disease from the index case.	Individuals resident in households within 1 km radius of the residence of the index case.
3.	Proactive Case Detection (PACD)	Screening high risk population unrelated to an index case of malaria.	
	PACD travel-cohorts	Travellers from a malaria endemic country with shared characteristics, unrelated to an index case.	Members of a group of military personnel returning from a UN Peacekeeping Mission in a malarious country; a group of post-war refugee returnees from India; Gem trader communities who frequently travel to Africa
	PACD spatial-cohorts	Population groups, either Sri Lankan nationals or foreigners from an endemic country who are resident in Sri Lanka in previously malarious and highly receptive areas (i.e. having a high prevalence of the principle vector *An.culicifacies*).	Populations living in previously endemic areas without a history of recent travel; Groups of foreign labour from malaria endemic countries temporarily resident in receptive areas; Refugees from malaria endemic countries

*Households within a radius of 1 km from the residence of the index case

This study analyses and presents the outcomes of the WHO-recommended malaria case surveillance strategies used in Sri Lanka over a three-year period from 2017 to 2019, to examine yields from each of them in terms of detecting malaria infections.

## Methods

### Case surveillance strategies

Three main case surveillance strategies recommended by the WHO are used In Sri Lanka. They are:

(1) Passive Case Detection (PCD) which is the detection of malaria cases among patients who on their own initiative seek health care usually for fever. (2) Reactive Case Detection (RACD) which is surveillance conducted in response to a case of malaria (referred to as the “index case”) when detected. This includes contact tracing and screening all those who may have had the same exposure to malaria as the index case, such as those who travelled with the index case (these will be referred to as “travel-cohorts”). RACD also includes screening people who were resident in the vicinity of the residence of the index case who may, therefore, have contracted malaria either from the same source as the index case, or by transmission from the index case (these will be referred to as “spatial-cohorts”), i.e., residents of all houses in the area within 1 km radial distance from the residence of an index case. The rationale for this is first, if the malaria infection of the index case was locally acquired (i.e. an indigenous case), there could be others in the area who may have been infected by the same source of infected mosquitoes, and for this the screening is conducted within two days of detecting a case, and is referred to as primary RACD screening. Second, irrespective of whether the index case was locally acquired or imported, he/she could have infected mosquitoes prevalent in the area prior to being diagnosed and treated, and thereby the infection could have been transmitted to others in the area. To detect such infections the screening of the neighbourhood community, i.e. residents of all houses within 1 km radius of the residence of the index case (referred to as secondary RACD screening) is carried out 3–4 weeks after the detection of the index case allowing sufficient time for the parasite’s mosquito cycle and the incubation period in the next infected person to be completed. These two types of primary and secondary RACD screening of residents of the area are in compliance with WHO recommendations [2] and has been conducted routinely for every case of malaria detected in the country depending on the circumstances. (3) Proactive Case Detection (PACD) which is the screening of high-risk populations not prompted by a case of malaria. For example, those who have travelled as a group from a malaria endemic country (referred to as “travel-cohorts”), or are resident in previously malarious areas or areas of high receptivity to malaria (referred to as “spatial-cohorts”). When such a cohort is identified, all members of the cohort are screened. These methods are described with examples in Table 1.

## Data sources and analysis

The national malaria database on case surveillance maintained by the Anti Malaria Campaign (AMC), which is a compilation of data from all districts of the country, was used in this analysis. Data was also extracted from individual case reports of malaria patients when required for more detailed examination of aspects not captured in the national database. Given that the case surveillance reporting and recording system has evolved over the years since elimination, the analysis was confined to the three most recent years (i.e.2017–2019) as they would be most representative of the current case surveillance system.

Over the past 7 years since elimination, 30–40% of malaria patients reported in the country were those in whom malaria was suspected and/or detected in the private health sector [7]. It is the practice that when a private hospital, clinic or laboratory suspects or detects a case of malaria, the AMC Headquarters or Regional Malaria Offices are informed, and it is the personnel of the AMC who then confirms the malaria diagnosis, provides the medicines free-of-charge and follows up the patient thereafter in accordance with the National Programme’s case management guidelines [8]. All case detections made primarily in the private sector have thus been captured in the AMC database under the category of Passive Case Detection.

This analysis has been based on the number of blood smears examined microscopically as the measure of the volume of tests conducted. The principal method of diagnosing malaria in Sri Lanka has been, and is, microscopic examination of blood smears. Rapid Diagnostic Tests (RDTs, (Carestart™) are being used in some specific situations, such as in hospitals if treatment of a patient needs to be started urgently when microscopy services are not available after regular working hours. However, even in such situations if RDTs are used as a screening tool for population groups, blood smears are prepared and subjected to microscopy at a later time. Therefore, any RDT performed has been captured under blood smears in the data used for this analysis.

Data was entered into MS Excel and analysed using SPSS (version 23). The descriptive statistics were generated using SPSS and the charts were developed in Excel. The yield of a particular case surveillance method was calculated as follows:

(Number cases detected by method) / (Number slides examined by method) x 100,000.

The yield was expressed per 100,000 blood smears examined. Comparisons were made between the different surveillance methods and among years.

A detailed analysis of sub categories of case surveillance methods was confined to the most recent year 2019. The reason being that whilst data on the three main case surveillance strategies, PCD, RACD and PACD, are available for each of the past 3 years, further disaggregated data on the number of blood smears examined in sub-categories within these strategies (for example, within RACD surveillance data on travel contacts of an index case versus those on neighbouring households of an index case) (Table 1) was only available for 2019. This is because the data entry system prior to 2019 was designed to capture the blood smears examined under the broad categories of PCD, RACD and PACD and not under the two sub-categories.

## Results

During the 3-year period 2017–2019, 1.09, 1.12 and 1.16 million blood smears have been examined microscopically respectively each year by the Anti Malaria Campaign. Nearly 40 % of these blood smears were for the yearly routine screening of blood donors conducted for the National Blood Bank (Table 2). Since malaria was eliminated in 2012, not a single sample from blood donors has been positive for malaria. These blood donor-smear examinations are conducted by the AMC on the request of the National Blood Bank to comply with the Blood Bank’s guidelines for blood safety, rather than as a core surveillance mechanism for the AMC. Therefore, this category of smear examinations has been excluded from the analysis below. Thus, excluding blood donors’ smears, 666,325, 683,626 and 725,149 blood smears have been examined microscopically by the AMC for malaria case surveillance in 2017, 2018 and 2019, respectively (Table 2). In years 2017, 2018 and 2019 the number of malaria patients detected in the country has been 57, 48 and 53, respectively (Table 3). All these were imported infections except for a single case of “introduced” malaria in 2018 [4].

**Table 2:** Blood smears examined by AMC, 2017–2019

Year	Number (%) of blood smears examined for		Total blood smears examined
	Blood Bank	Malaria surveillance	
2017	422,965 (38.8)	666,325 (61.2)	1,089,290
2018	445,444 (39.5)	683,626 (60.5)	1,129,070
2019	439,765 (37.8)	725,149 (62.2)	1,164,914

**Table 3:** Performance of all three case surveillance strategies PCD, RACD and PACD in terms of the number of malaria infections detected, 2017–2019

Year		No. of blood smears by each case surveillance strategy		
		PCD*	RACD*	PACD*
2017				
Positive		56	1	0
Examined		289,495	3,085	373,745
2018				
Positive		48	0	0
Examined		298,072	10,509	375,045
2019				
Positive		47	3	3
Examined		352,313	22,248	350,588
All years				
Positive		151	4	3
Examined		939,880	35,842	1,099,378
Yield per 100,000 smears		16.1	11.2	0.3

* PCD-Passive Case Detection; RACD-Reactive Case Detection; PACD-Proactive Case Detection

## Passive case detection

**Fig. 1:**
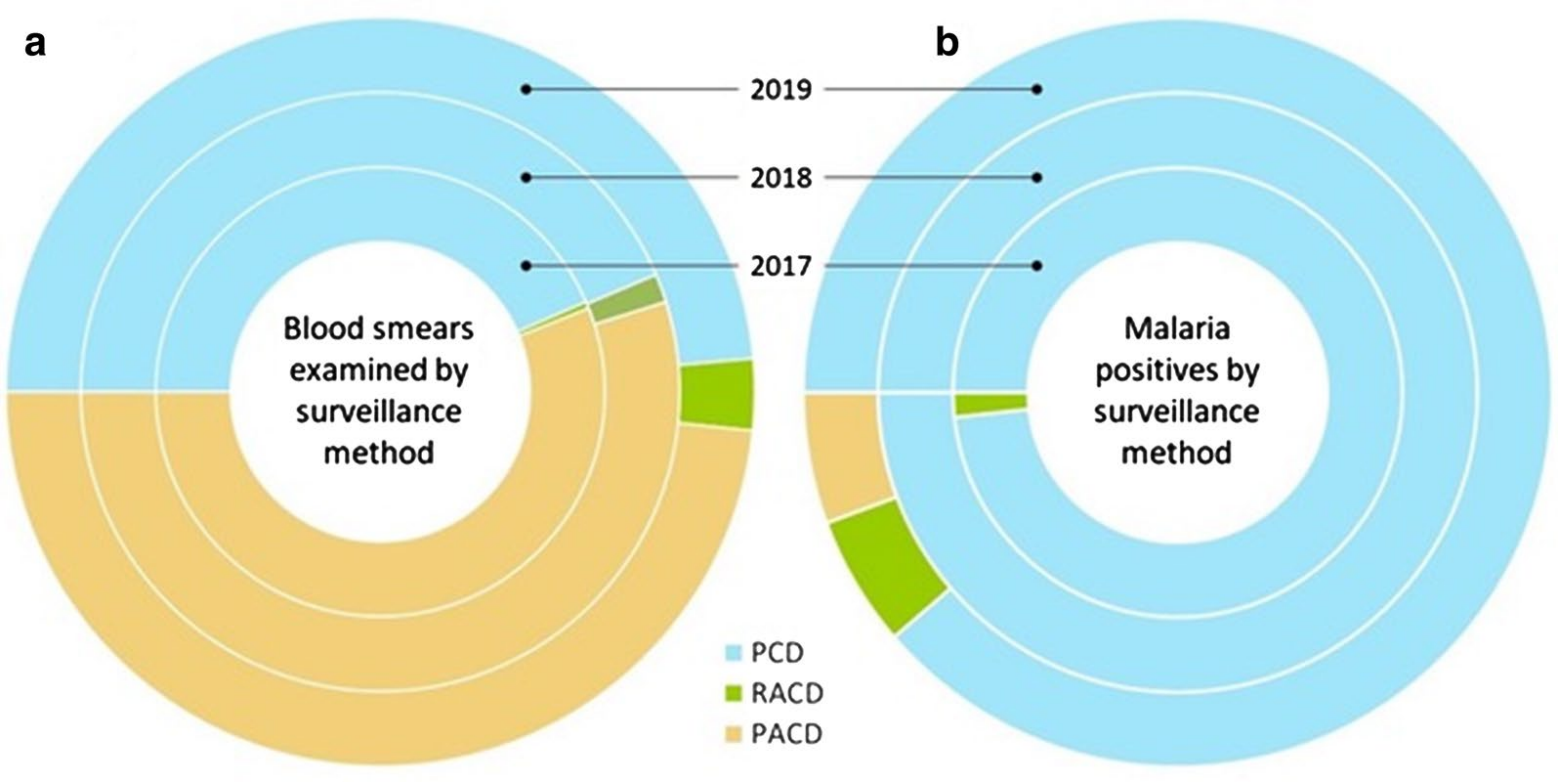
Blood smears examined (**a**) and positive (**b**) for malaria by category of case detection 2017–2019. The proportion of blood smears examined (**a**) and positive (**b**) for malaria by PCD, PACD and RACD each year, 2017–2019. Each circle represents a single year, and the different colors each represent the proportion of blood by the different case surveillance strategies

**Fig. 2:**
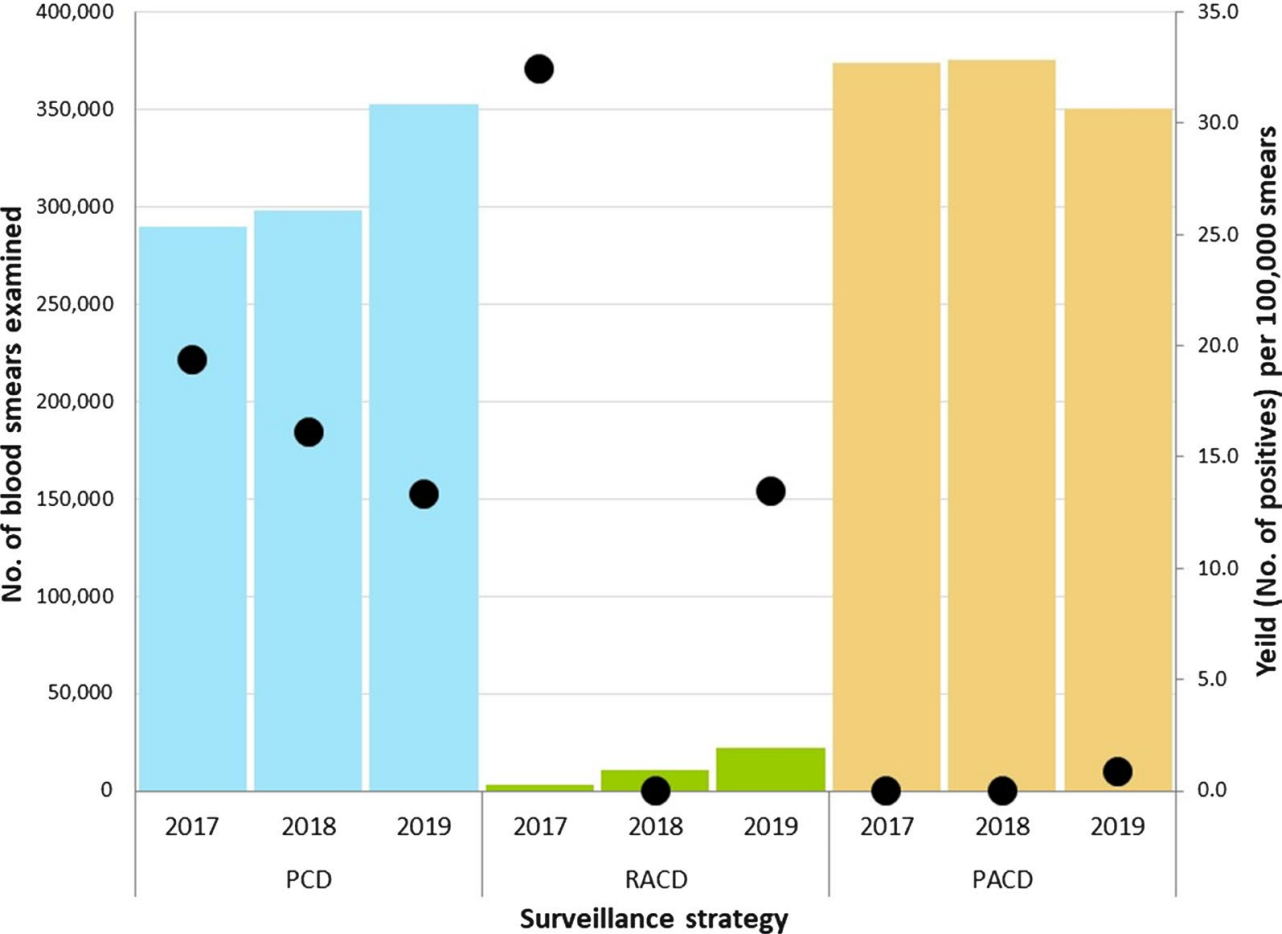
Number of blood smears examined for malaria by case surveillance strategies during the period 2017–2019 and the yield of positives from each strategy. The number of blood smears examined for malaria by case surveillance strategies, PCD (blue), RACD (green), and PACD (ochre) (columns) during 2017, 2018 and 2019 and the yield of positives per 100,000 smears (dots) from each strategy in each year

Nearly half (43.4−48.6%) of all blood smears examined by the AMC for malaria surveillance from 2017 to 2019 were those requested by health care providers in the public and private health sectors for diagnosing patients presenting with symptoms suggestive of malaria, i.e. PCD (Fig. 1a; Table 1). A vast majority of malaria patients in the country – 98, 100 and 89% in years 2017, 2018 and 2019 respectively - were detected through this Passive Case Detection mechanism (Table 3). Of 158 malaria infections reported in the country during the 3-year period, 151 infections were detected through PCD, and only 4 and 3 cases were detected through RACD and PACD respectively (Figs. 1 and 2). The yield of malaria positives by PCD was 16.1 positives per 100,000 blood smears examined (Table 3; Figs. 1 and 2).

## Active case detection

Active Case Detection (ACD) the other major strategy for malaria case surveillance in the country comprises two distinct categories namely Reactive Case Detection (RACD) and Proactive Case Detection (PACD) (Table 1).

## Reactive case detection (RACD)

Only 1.7 % of blood smears examined during the 3 years were from the RACD strategy but it gave as nearly high a yield as Passive Case Detection—i.e. 11.2 positives per 100,000 blood smears examined (Figs. 1 and 2; Table 3). This method comprised two distinct categories (Table 1).

1) Screening of “travel-cohorts” of an index case – e.g., those who have had the same or similar exposure as the index case, such as co-travellers from an endemic country. In 2019, all three positives detected through RACD belonged to this category. However, in the past 3 years (and in fact, since malaria elimination in 2012) not a single positive case has been detected by either primary or secondary screening of residents of neighbourhoods within the RACD strategy.

In the year 2019, tracing of travel contacts and screening them which constituted only 1.7 % of blood smears collected by RACD, gave an extremely high yield of detection of 806.5/100,000 blood smears, whereas screening of residents within the neighbouring area of an index case has given a zero yield in all three years, despite it constituting as much as 98.3 % of the RACD blood smears (Table 4).

**Table 4:** Outcomes of PCD, and disaggregated RACD and PACD in 2019

Case surveillance (sub) categories		No. of blood smears			Yield per 100,000 smears
		Examined	(%)	Positive	
PCD		352,313	(48.6)	47	13.3
RACD					
Travel-cohorts		372	(0.1)	3	806.5
Spatial-cohorts*		21,876	(3.0)	0	0
PACD					
Travel-cohorts		6,688	(0.9)	3	44.9
Spatial-cohorts		343,900	(47.4)	0	0

* Primary and secondary screening of residents of households within 1 km radius from the residence of the index case

## Proactive case detection (PACD)

As much as 53% of all blood smears examined in the three years were from the PACD strategy of case surveillance (Figs. 1 and 2). However, PACD gave a very low, and the lowest yield of detection of malaria (n = 3) amongst all case surveillance strategies – i.e. 0.3 cases per 10,000 blood smears (Table 3). PACD in 2017 and 2018 did not yield any cases. Two broad categories of people are being subjected to screening by PACD (Table 1). (1) Travel cohorts – meaning travellers with shared characteristics, but unconnected to an index case. (2) Spatial cohorts – groups of people who are living in previously malarious and highly receptive areas in Sri Lanka i.e. in areas with a high malariogenic potential.

In 2019 (for which year disaggregated data was available), all three patients detected through PACD were in the “travel-cohort” category, the screening of which accounted for only 0.9% of all PACD smears. Thus, the screening of “travel-cohorts” gave a high yield of 44.9 positives per 100,000 smears. The other category of PACD which screened residents who could be at risk owing to their residence in previously malarious areas or in areas currently having a high receptivity which accounted for 98% of the 350,588 PACD smears examined in 2019 gave a zero yield (Table 4).

## Discussion

Of the three principal case surveillance strategies deployed by the AMC over the past three years, the highest proportion of cases detected (n = 151; 95.6%), and also the highest yield of positive cases (16.1 positives per 100,000 persons tested), has been from Passive Case Detection. The number of cases detected by both RACD and PACD over the 3 years were small being four and three cases, respectively. However, Reactive Case Surveillance gave a much higher yield of 11.2 positives per 100,000 smears, than PACD which gave a mere 0.3 positives per 100,000 smears examined. Much fewer smears were collected and examined by RACD (n = 35,842) than by PACD (n = 1,099,378) which accounts for this difference in yield. These findings have been consistent over the three-year period examined here.

RACD and PACD each comprise of some very distinct sub-categories as explained in Table 1. Greater insights into the yields from different case surveillance strategies emerge from a sub-analysis of RACD and PACD, which was performed only for the most recent year on account of the data recording system not accommodating the classification in previous years. Thus, in 2019 within RACD, the tracing and screening of travel-cohorts (travel contacts of index cases) was the highest yielding of all strategies (806.5 positives per 100,000 persons screened). Within the PACD strategy the screening of “travel-cohorts” of returnees to the country unconnected to an index case, and identified as being at high-risk also gave a very high yield (44.9 positives per 100,000 persons screened). Thus, collectively three categories gave extremely high case yields: (1) Passive Case Detection; (2) screening of travel-cohorts in relation to an index case in RACD; and (3) screening of travel cohorts within PACD unrelated to an index case. They accounted for nearly half (49.6%) of blood smears screened in 2019. Importantly, all of the malaria cases reported during the entire three year period of this study were detected through these three case surveillance strategies.

The rest constituted just over half of the blood smears examined (50.4% of the total) in 2019. And these strategies gave a zero yield of cases over the three years. These were the Reactive screening of spatial cohorts, i.e. residents of houses located in the vicinity of an index case which constituted 3% of all blood smears examined and the Proactive screening of spatial cohorts—i.e. population groups, of either foreign or Sri Lankan nationality resident in Sri Lanka (with or without a history of travel overseas), who were considered to be at risk because they live in previously malarious areas and/or areas with a high degree of receptivity, which constituted 47.3% of blood smears examined in 2019.

The two broad case surveillance strategies for malaria, passive and active case surveillance, engage two very different arms of the health system. PCD entails operating within the existing curative sector of the health system, in both the private and public sectors, and requires regular and continuous information to be provided to clinicians on the need to test for malaria in febrile patients with a travel history. It also entails strengthening the diagnostic services for malaria throughout the health care system in both private and public sectors to ensure that patients have wide access to a high quality malaria diagnosis. These are very challenging activities when the malaria disease burden is extremely low, and for a disease rarely encountered by clinicians as is the case in Sri Lanka at present [9–13].

Contrastingly, both RACD and PACD requires extensive community level operations, and human and other resources in order to investigate index cases and trace their contacts especially in the case of RACD. PACD needs community intelligence on where high-risk groups may reside, and entails actively searching for and screening them. These activities require a community workforce and constant vigilance on information from the field. In Sri Lanka it is public health inspectors and field staff of the Regional Malaria Offices supervised by the Regional Malaria Officers that perform these tasks. These strategies also require transport facilities, and entail close collaborations with other sectors beyond health—immigration, airport and aviation and port health, the military, police, tourism, as well as departments dealing with repatriation, refugees, and special categories of travellers such as pilgrims [5, 14–16].

Although both strategies ACD and PCD are beset with challenges in a post-elimination situation, PCD is an inherent component of the health information system, and this analysis confirms its critical role in surveillance. Because, firstly, from an ethical standpoint PCD responds to individuals with illness. It is therefore indispensable. Secondly because it is a very effective case surveillance strategy – 95.6% of the malaria cases during the 3 years of study was detected through this strategy. The yield of 16.1 per 100,000 blood smears examined was also high, making PCD an effective strategy. The need to strengthen it as a major surveillance strategy is obvious, and the effort may need to be a continuous one, post-elimination [9–13].

A recent publication reviewed RACD strategies in endemic countries examining the screening of spatial cohorts within RACD. It reported that none of the published studies had compared the effectiveness or cost-effectiveness of RACD in elimination settings *vis*-*a*- *vis* no RACD, meaning that effectiveness data was not available [17]. Several of the documented descriptions of RACD in houses neighbouring an index case that were reviewed [17] were in countries which were approaching elimination and where transmission was still ongoing even at a low intensity and therefore, not surprisingly, many of them reported finding cases through RACD spatial cohorts [18–22]. The study reported here might, therefore, be the first to examine the yield, of not only RACD but of all other case surveillance strategies as well.

These findings must be interpreted within the context of the following limitations. The analysis was based on secondary data, generated by the AMC’s routine data collection system and therefore, subjected to the data quality assurance methods of the institution and not by the investigators. However, the relevance of this study is also that it is based on operational data routinely collected by the national programme and is therefore highly representative of program activity and is generalizable. Secondly, the separation of active case surveillance data into travel and spatial cohorts has been only presented for a single year, 2019. This is because the number of blood smears examined was not categorized into these subdivisions in previous years. However, the AMC’s records show that no malaria cases were detected by screening spatial cohorts within either RACD or PACD in 2017 and 2018 either. Therefore, the finding that spatial cohorts give a poor yield has validity more broadly for the entire period of this study.

Effectiveness of case surveillance strategies, in terms of the yield of positives, cannot be the sole criterion for making policy decisions on their continued use to prevent the re-establishment of malaria. For example, the programme could ill afford to have missed the relatively small number of cases detected by RACD and PACD in the past three years. If these operations had not been performed, the seven cases may not have been detected because the infected persons had no symptoms, or their detection may have been long delayed resulting in onward transmission until, possibly an outbreak occurred. Besides, the usefulness and even effectiveness of these strategies could change with circumstances. For example, although the screening of spatial cohorts i.e. neighbouring houses of index cases did not lead to the detection of any cases over the seven years since elimination (data not shown for the years 2012–2016), it may be relevant in situations where an introduced or indigenous case of malaria has been reported in a highly receptive area, making a malaria outbreak imminent.

The data here shows that within RACD, reactive screening of travel cohorts who are contacts of an index case, and within PACD, the screening of travel cohorts arriving from endemic countries give extremely high yields, even though only a few (4.4% of all cases) were detected through these two strategies over the three years. The high yield in these strategies owes to the relatively focused screening of a small numbers of people who are at high risk. More importantly these findings call to question the effectiveness of two other categories of case surveillance operations which are continued unmodified since WHO certification, and are being performed routinely, accounting for about half of all blood smears examined. These are the routine screening of spatial cohorts, i.e. of neighbourhood households of an index case within RACD, and within PACD the intermittent screening of populations groups residing even temporarily in previously endemic regions unrelated to an index case and regardless of whether they had recently travelled overseas. The findings described here also imply that the routine and intermittent screening of population sub-groups, merely because the receptivity of the area is high, and without any other risk factor being present in the area, may be wasteful. This is all the more significant given that just over half of the smears examined were from these two categories, mainly from the latter.

Over the past three years the screening of travel cohorts within both RACD and PACD gave high yields. The screening travel-cohorts in both reactive and proactive screening as suggested by this study becomes even more important because of the current post-war development boom in Sri Lanka. This has led to large numbers of migrant worker populations, business travellers and tourists entering the country, which has considerably increased the risk of importing malaria [4, 23]. Equally important is the finding that the screening of spatial cohorts in either category led to hardly any case detections. These findings call for identifying more discerning criteria with which to narrow the selection and sizes of risk populations for screening in post-elimination settings. Moreover, one could argue that case surveillance criteria need to be refined not only on the basis of a case classification e.g., imported, indigenous, introduced case, but also taking into consideration the broader contexts – including the receptivity of the area, the duration of time that lapsed between the onset of illness and treatment, and parasite species – which influence the probability of onward transmission.

This study highlights the importance of regular analysis and review of country data post-elimination to inform the programme on which case surveillance strategies to invest in. This analysis may also serve to provide evidence for refining existing policy guidelines on case surveillance in the POR phase of malaria in Sri Lanka and other countries after elimination. The optimal use of case surveillance strategies will help ensure that government allocated funds for malaria which, after elimination tend to be limited [24], are used more effectively.

## Conclusions

In the POR phase, PCD is the most effective surveillance mechanism and should be strengthened and remain as the main malaria surveillance strategy. When Reactive and Proactive Case Detection methods focussed on travel cohorts the yield of cases was very high, implying that there should be a greater focus on travel cohorts within active case surveillance. The screening of spatial cohorts within Reactive and Proactive Case Detection on a routine basis, and solely because people are resident in previously malarious areas appears to be wasteful, except in situations where the risk of local transmission is very high, or is imminent.

## Data Availability

The datasets generated and/or analysed in this publication are not publicly available due to the fact that they belong to the Ministry of Health, Sri Lanka. Clarifications regarding data can be made through Dr. Prasad Ranaweera, Director of the Anti Malaria Campaign, Sri Lanka who is an author of this publication.

## Abbreviations

ACD: Active Case Detection
AMC: Anti Malaria Campaign
PACD: Proactive Case Detection
PCD: Passive Case Detection
POR: Prevention of re-establishment
RACD: Reactive Case Detection
RDT: Rapid Diagnostic Tests
RMO: Regional Malaria Officer
WHO: World Health Organization
